# Brendel Modification of Wassermann Test

**Published:** 1913-09

**Authors:** 


Brendel Modification of Wassermann Test Ward Burdick of Denver, *Col. Med.,* April, 1913, (cf. Brendel & Miller, *Münch. Mad. Woch.,* August, 1912), reports a series of 100 Brendel tests, 13 negatives, 87 positive, all controlled as perfectly as possible by other signs and apparently all reliable, that is, the positive reactions occurring in syphilis, the negative in non-syphilitics. The test, however, cannot be used independently of the original Wassermann.

